# The Institutional Library

**Published:** 1913-06-28

**Authors:** 


### THE INSTITUTIONAL LIBRARY.

The Practitioner's Encyclopædia of Medicine and Surgery in all their Branches. Edited by J. Keogh Murphy, M.C., F.R.C.S. Second Edition. (London: Henry Frowde and Hodder and Stoughton. 1913. Pp. 1,445. Illustrated. Price 35s.)

The passion for encyclopædias is deeply rooted in human nature. The prevalent desire to possess handbooks or massive tomes containing information on every branch of knowledge has been exploited more or less successfully for a long time: witness the innumerable "reference" books now on the market, from sixpenny year-books to costly encyclopædias "given away" with mahogany bookcases. That this principle has been already extended to limited spheres of medicine is not a matter for surprise, but that an attempt should be made to present the *whole* art of general practice in one comprehensive volume must be regarded as an exceedingly bold venture. Yet this is what this "Practitioner's Encyclopædia of Medicine and Surgery" purposes to do. Not only does the field covered include medicine in its usually accepted sense, but it also embraces general and special surgery, midwifery and gynæcology, mental disorders, anæsthetics, eye, ear, throat, nose, and skin diseases, besides many other special topics, such as medico-legal points and physical exercises. It may well be wondered how articles on all these subjects can be at the same time sufficiently explicit to be of practical value and yet sufficiently brief to be compressed into one volume which is not too unwieldy in size. However impossible this may at first sight appear, it must be conceded that the task has been accomplished by the editor and his numerous colleagues with more success than we were originally inclined to expect. The book, in fact, contains a good deal more letterpress than one would imagine it does from its size. To begin with, there are well over fourteen hundred double-column pages of thin paper; the leaves are, happily, easily turned, and the illustrations or the type on the other side do not disturb the eye. Then, again, anatomical and pathological details have been cut down to the barest limits, except where they are of practical application, in spite of the contradictory sentence in the preface—a meaningless sentence which has escaped correction in this the second edition. Though naturally it has been impossible to present in full all the views which are at present current concerning certain debatable topics, notably in the section on general medicine, still this encyclopædia is indeed a mine of information for the general practitioner. A glance at the table of contents is not uninteresting. It discloses the fact that the editor has been at some pains to secure the collaboration of recognised experts for the innumerable sections and subsections. If the list of the one hundred and sixty odd contributors does not include always the names of the most eminent specialists in the several branches of medicine, surgery, and midwifery, on the whole the articles are more than usually well abreast of the times. The difficulties of a task of such magnitude as this encyclopædia represents are only known to the unfortunate editor, and as far as such a work can do so this comprehensive volume does for the moment justify the editor's claim to contain "articles which convey the total information in the most succinct and most definite manner."

The continual repetition of contributors' appointments in the table of contents is not only irritating but totally unnecessary, seeing that it spreads out the lists to an inconvenient length, and there is another separate reference list giving again all these painful and possibly ethical advertisements. General practitioners do sometimes possess a copy of the "Medical Directory." Obviously it is impossible in the space at our command to refer in any detail to the individual efforts of such a crowd of contributors, and it would be manifestly unfair to single out special articles without the justification of having read the whole book from cover to cover. This we make no pretence at having done; but we have been at some pains to test this encyclopædia in the manner in which it is intended it shall be tested by the general practitioner—namely, by looking up points on very varying subjects that crop up in the course of an ordinary and quite "general" practice. The conclusion arrived at is that this book does fulfil its purpose. Whether on ear work or midwifery or psychology or plain medicine, there is available in most cases sufficient guidance for the general practitioner's purpose. On certain details a few practitioners may require more precise information, but these men are the exceptions, and they, too, know how to get what they need; the majority of medical men would certainly never need to go farther on their own responsibility. It is for this large majority that this volume will be of practical service. A gratifying feature, and one for which the editor is presumably responsible, is the directness of the "attack" which characterises the articles; no space is wasted in introductory remarks, and "essays" were evidently at a discount. As regards the arrangement of the book it may be said that the "systemic" rather than the "alphabetical" order has fully justified its choice, and under sections familiar to the practitioner he will find it easy to refer to any subject, whilst the full index provides a large number of cross-references. Such are very necessary to supplement the table of contents, for we note that under the subheading "Diseases due to Vegetable Parasites" there is described only one—actinomycosis—and the reader must dive into the Skin Diseases section to find sporotrichosis, etc. A certain amount of repetition has been unavoidable owing to the systemic arrangement—such diseases as tuberculosis and syphilis being treated in several sections; but apart from the necessary compression due to the nature of the work this systematic arrangement is rather advantageous than otherwise. This second edition differs from the first one chiefly in the inclusion of the addenda of that issue in their proper places and the addition of several new articles, including Dr. Mackenzie's contribution on Angina Pectoris.

The Biology of Tumours. By C. Mansell Moullin, M.D., F.R.C.S., Consulting Surgeon to the London Hospital. (London: H. K. Lewis. 1913. Pp. 39. Price 2s. net.)

This slender volume is the Bradshaw Lecture recently delivered before the Royal College of Surgeons by a distinguished ex-member of the London Hospital surgical staff. In it Mr. Mansell Moullin is content to deal with established observations and reasonable inferences from them, without diverging too much into hypothetical considerations or wild speculation. If there is nothing of very outstanding novelty or originality in this review of knowledge, it is at any rate a useful thing to take stock of the latest research every now and then; and this Bradshaw Lecture well maintains the traditions of the series of these annual orations.

